# Perioperative Complications and In-Hospital Mortality After Radical Prostatectomy in Prostate Cancer Patients with a History of Heart Valve Replacement

**DOI:** 10.3390/jcm14145035

**Published:** 2025-07-16

**Authors:** Natali Rodriguez Peñaranda, Carolin Siech, Letizia Maria Ippolita Jannello, Francesco Di Bello, Mario de Angelis, Jordan A. Goyal, Fred Saad, Shahrokh F. Shariat, Nicola Longo, Alberto Briganti, Ottavio de Cobelli, Felix K. H. Chun, Stefano Di Bari, Ivan Matteo Tavolini, Stefano Puliatti, Salvatore Micali, Pierre I. Karakiewicz

**Affiliations:** 1 Cancer Prognostics and Health Outcomes Unit, Division of Urology, University of Montréal Health Center, Montreal, QC H2X 0A9, Canada; siech@med.uni-frankfurt.de (C.S.); letizia.jannello@unimi.it (L.M.I.J.); francesco.dibello@unina.it (F.D.B.); deangelis.mario@hsr.it (M.d.A.); jordan.goyal@umontreal.ca (J.A.G.); fred.saad@umontreal.ca (F.S.); pierrekarakiewicz@gmail.com (P.I.K.); 2 Department of Urology, AOU di Modena, University of Modena and Reggio Emilia, 41126 Baggiovara, Italy; 297009@studenti.unimore.it (S.D.B.); stefano.puliatti@unimore.it (S.P.); salvatore.micali@unimore.it (S.M.); 3 Department of Urology, University Hospital, Goethe University Frankfurt, 60590 Frankfurt am Main, Germany; felix.chun@ukffm.de; 4 Department of Urology, IEO European Institute of Oncology, IRCCS, 20141 Milan, Italy; ottavio.decobelli@ieo.it; 5 School of Medicine, Università degli Studi di Milano, 20133 Milan, Italy; 6 Department of Neurosciences, Science of Reproduction and Odontostomatology, University of Naples Federico II, 80131 Naples, Italy; nicola.longo@unina.it; 7 School of Medicine, Vita-Salute San Raffaele University, 20132 Milan, Italy; briganti.alberto@hsr.it; 8 Division of Experimental Oncology/Unit of Urology, Urological Research Institute (URI), IRCCS Ospedale San Raffaele, 20132 Milan, Italy; 9 Department of Urology, Comprehensive Cancer Center, Medical University of Vienna, 1090 Vienna, Austria; shahrokh.shariat@meduniwien.ac.at; 10 Department of Urology, Weill Cornell Medical College, New York, NY 10021, USA; 11 Department of Urology, University of Texas Southwestern Medical Center, Dallas, TX 75390, USA; 12 Hourani Center for Applied Scientific Research, Al-Ahliyya Amman University, Amman 19328, Jordan; 13 Department of Oncology and Haemato-Oncology, Università degli Studi di Milano, 20122 Milan, Italy; 14 IGG Italian Germ Cell Cancer Group, 16122 Genova, Italy; i.tavolini@ausl.pc.it

**Keywords:** comorbidities, mortality, heart surgery, heart valve replacement, NIS, prostate cancer

## Abstract

**Objective:** To test for in-hospital mortality and complication rates in a population-based group of patients with vs. without a history of heart valve replacement undergoing radical prostatectomy (RP). **Methods:** Relying on the National Inpatient Sample (2000–2019), prostate cancer patients undergoing RP were stratified according to the presence or absence of heart-valve replacement. Multivariable logistics and Poisson regression models addressed adverse hospital outcomes. **Results:** Within the NIS, 220,358 patients underwent RP. Of those, 694 (0.3%) had a history of heart valve replacement. The patients undergoing heart valve replacement were older (median age 66 vs. 62 years). The proportion of patients with a history of heart valve replacement increases with the Charlson Comorbidity Index (CCI): CCI 0–0.3%, CCI 1–0.4%, and CCI ≥ 2–0.7%. Patients with a history of heart valve replacement exhibited higher rates of postoperative bleeding (<1.5% vs. <0.1%; odds ratio (OR) 16.2; *p* < 0.001), cardiac complications (7.5% vs. 1.2%; OR 3.9; *p* < 0.001), infections (<1.5% vs. 0.1%; OR 3.7; *p* = 0.01), critical care therapy (CCT) use (<1.5% vs. 0.4%; OR 2.5; *p* = 0.003), intraoperative complications (8.8% vs. 4.1%; OR 1.9; *p* < 0.001), transfusions (11% vs. 7.2%; OR 1.5; *p* < 0.001), longer hospital stay (mean 3.39 vs. 2.37 days; rates ratio [RR] 1.4; *p* < 0.001), and higher estimated hospital cost (median 33,539 vs. 30,716 $USD; RR 1.1; *p* < 0.001). Conversely, no statistically significant differences were observed in vascular complications (*p* = 0.3) or concerning in-hospital mortality (*p* = 0.1). **Conclusions:** After RP, patients with a history of heart valve replacement exhibited a higher rate of eight out of nine adverse in-hospital outcomes. However, these differences did not translate into higher in-hospital mortality.

## 1. Introduction

Radical prostatectomy (RP) is one of the most widely used treatment options for localized prostate cancer [1,2]. As the population ages, an increasing proportion of prostate cancer patients present with significant cardiovascular comorbidities. Among them, individuals with a history of heart valve replacement represent a unique and understudied subgroup who may still require curative surgical intervention. However, perioperative management in these patients is often complex given the frequent need for chronic anticoagulation and their inherently elevated cardiovascular risk profile. This concern is reinforced by prior evidence showing that cardiovascular comorbidities such as atrial fibrillation and coronary artery disease are associated with increased perioperative morbidity in patients undergoing RP [3,4,5]. Although higher intraoperative and postoperative complications as well as higher risk of in-hospital mortality are intuitively expected in RP patients with heart valve replacement, it is not known how pronounced such an increase might be. Indeed, no published reports addressing patients with a history of heart valve replacement who underwent radical prostatectomy have appeared in the modern English language medical literature. Recent studies have suggested that RP may offer better cardiovascular safety compared to radiotherapy in prostate cancer patients with pre-existing heart disease, particularly among the elderly [6]. Others have shown that cardiovascular patients undergoing RP may be more vulnerable to infectious or cardiac complications [3], further supporting the clinical importance of this topic.

We addressed this knowledge gap and hypothesized that perioperative complication and in-hospital mortality rates after RP do not differ between patients with vs. without a history of heart valve replacement. To test this hypothesis, we relied on a large-scale population-based database (National Inpatient Sample database 2000–2019) of prostate cancer patients. The National Inpatient Sample (NIS) is the largest publicly available all-payer inpatient healthcare database in the United States. It includes data on patient demographics, diagnoses, procedures, hospital charges, length of stay, and hospital characteristics. The NIS is designed to approximate a 20% stratified sample of all discharges from U.S. community hospitals, enabling population-level estimates [7].

## 2. Materials and Methods

### 2.1. Data Source

Relying on discharge data from the National Inpatient Sample (NIS 2000–2019) [3], perioperative complications, length of stay, estimated hospital cost, and in-hospital mortality after RP were assessed. All diagnoses and procedures were coded according to previously published methodology [8,9,10].

### 2.2. Study Population

Patients aged ≥18 years with a primary diagnosis of prostate cancer (ICD-9-CM code 185 and ICD-10-CM code C61) and treated with RP (ICD-9 codes 604, 605, 606.2, 606.9, and 607.3 and ICD-10-PCS codes 0V500ZZ, 0VT00ZZ, and 0VT30ZZ) were included [11]. Primary stratification relied on a history of heart valve replacement (ICD-9-CM codes V42.2 and V43.3 and ICD-10-CM codes Z95.2-Z95.4) [12]. Patients with missing or invalid procedure codes for radical prostatectomy and those with incomplete data on key variables or covariates of interest (e.g., age, Charlson Comorbidity Index, and outcomes of interest) were excluded from the analysis. These criteria ensured consistency and completeness in the statistical models.

### 2.3. Definition of Variables for Analyses

Study endpoints included length of stay, estimated hospital cost, intraoperative and postoperative complications (bleeding, cardiac complications, vascular complications, infections, and transfusion) [13], in-hospital mortality, and use of Critical Care Therapies (CCT) as defined according to previously established methodology [14,15,16]. Estimated hospital cost was calculated relying on total hospital charges provided by the NIS [7]. All calculations were adjusted to 2019 United States dollar relying on the overall Consumer Price Index [17]. Comorbidities were assessed using the Deyo adaptation of the Charlson Comorbidity Index (CCI) [18] based on ICD coding algorithms described by Quan et al. [19]. Covariates consisted of patient characteristics including age at admission (years, continuously coded) and CCI (0 vs. 1 vs. ≥2), as well as hospital characteristics including hospital region (West vs. Midwest vs. Northeast vs. South) and teaching hospital status (teaching vs. non-teaching).

### 2.4. Statistical Analyses

Descriptive statistics were calculated for baseline characteristics and main outcomes. Categorical variables were summarized as frequencies and percentages, while continuous variables were reported as medians with interquartile ranges (IQR) or means with standard deviations (SD). Differences in continuous variables were tested using the Wilcoxon rank sum test, and differences in categorical variables were assessed with Pearson’s Chi-square test. Trends in Estimated Annual Percentage Change (EAPC) were evaluated using least squares linear regression. Univariable and multivariable Poisson regression models were applied to assess length of stay and hospital cost, while logistic regression models were used for perioperative complications and in-hospital mortality. All models accounted for clustering at the hospital level through generalized estimating equations (GEE) [15,16]. All multivariable models were adjusted for age at admission and comorbidities as assessed by the Charlson Comorbidity Index (CCI) to account for potential confounding factors between patient groups.

Analyses and reporting followed NIS reporting guidelines [7]. Due to NIS data reporting agreement, counts and associated proportions were reported as less than eleven for sample sizes of less than eleven patients. R software environment was used for statistical computing and graphics (R version 4.2.2; R Foundation for Statistical Computing, Vienna, Austria) [20]. All tests were two sided with a significance level set at *p* < 0.05.

## 3. Results

### 3.1. Descriptive Characteristics of the Study Population

**Figure 1:**
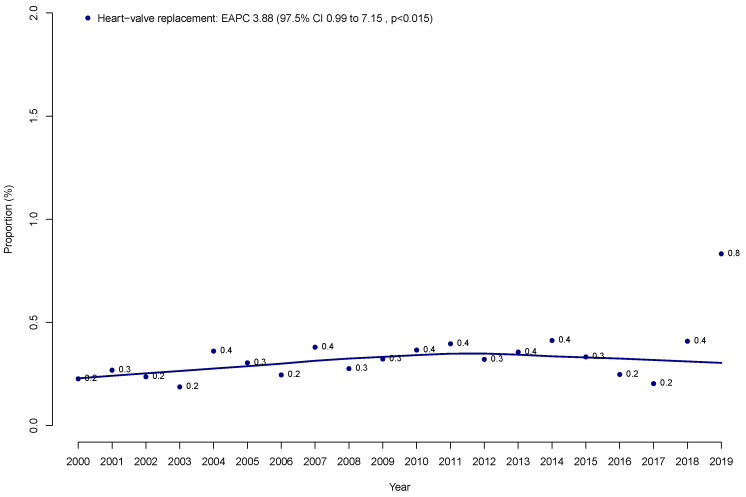
Proportion of prostate cancer patients who underwent radical prostatectomy with a history of heart valve replacement over time within the Nationwide Inpatient Sample (NIS) from 2000 to 2019.

Within the NIS (2000–2019), we identified 220,358 prostate cancer patients who underwent RP (Table 1). Of those, 694 (0.3%) had a history of heart valve replacement. Over time, the annual proportions of patients with a history of heart valve replacement increased from 0.2 to 0.8% in a statistically significant fashion (EAPC 3.88%, 97.5% confidence interval [CI] +0.99 to +7.15%; *p* < 0.002; Figure 1).

Patients with a history of heart valve replacement were older (median age 66 vs. 62 years; *p* < 0.001) than their counterparts without a history of heart valve replacement. The proportion of patients with a history of heart valve replacement increases with higher CCI scores: CCI 0–0.3%, CCI 1–0.4%, and CCI ≥ 2–0.7%. No differences in the proportion of patients with a history of heart valve replacement were recorded according to hospital region, hospital size, and teaching hospital status.

### 3.2. Perioperative Complications, Mortality, Length of Stay, and Cost Rates

Among the nine hospital adverse outcomes examined, RP patients with a history of heart valve replacement exhibited higher rates than their counterparts without a history of heart valve replacement. Specifically, RP patients with a heart valve replacement exhibited higher rates of cardiac complications (52 of 694 vs. 2739 of 219,664; 7.5% vs. 1.2%; *p* < 0.001; Table 2), intraoperative complications (61 of 694 vs. 9036 of 219,664; 8.5% vs. 4.1%; *p* < 0.001), infections (<11 of 694 vs. 221 of 219,664; <1.5% vs. 0.1%; *p* = 0.006), postoperative bleeding (<11 of 694 vs. 65 of 219,664; <1.5% vs. <0.1%; *p* < 0.001), blood transfusions (73 of 694 vs. 15,731 of 219,664; 11% vs. 7.2%; *p* < 0.001), and CCT (<11 of 694 vs. 873 of 219,664; <1.5% vs. 0.4%; *p* < 0.001). Conversely, RP patients with a history of heart valve replacement did not exhibit higher rates of vascular complications than their counterparts without a history of heart valve replacement (<11 of 694 vs. 561 of 219,664; <1.5% vs. 0.3%; *p* = 0.1). Most importantly, no statistically significant difference was recorded in in-hospital mortality between RP patients with heart valve replacement and without heart valve replacement (<11 of 694 vs. 126 of 219,664; <1.5% vs. <0.1%; *p* = 0.06). Finally, the mean length of stay was longer in RP patients with a history of heart valve replacement than their counterparts without a history of heart valve replacement (3.39 vs. 2.37 days; standard deviation [SD] 3.42 vs. 2.03 days; *p* < 0.001). Similarly, the median estimated hospital cost was higher in patients with a history of heart valve replacement than others (33,539 $USD vs. 30,716 $USD; interquartile range [IQR] 22,715, 51,178 vs. 20,796, 45,307 $USD; *p* < 0.001). For five of the eleven outcomes above, actual counts and actual proportions are not shown due to NIS reporting rules. Instead, counts of <11 and the associated proportions are shown.

### 3.3. Multivariable Logistic Regression Models

In multivariable logistic regression models, a history of heart valve replacement independently predicted higher rates of cardiac complications (multivariable odds ratio (OR) 3.92; 95% CI 2.89, 5.30 *p* < 0.001), intraoperative complications (OR 1.9; 95% CI 1.5, 2.5 *p* < 0.001), infections (OR 3.7; CI 95% 1.4, 9.8 *p* = 0.01), postoperative bleeding (OR 16.2; 95% CI 5.7, 46.3 *p* < 0.001), blood transfusions (OR 1.5; 95% CI 1.2, 1.8 *p* < 0.001), CCT use (OR 2.5; 95% CI 1.3, 4.6 *p* = 0.003), length of stay (rates ratio [RR] 1.4; 95% CI 1.3, 1.5 *p* < 0.001), and estimated hospital cost (RR 1.1; 95% CI 1.0, 1.2 *p* < 0.001). Conversely, no statistically significant differences were observed in vascular complications (*p* = 0.3) and in in-hospital mortality (*p* = 0.1) (Table 3).

## 4. Discussion

Although intuitively clinicians expect higher risk of complications in RP patients with a history of heart valve replacement, exact complication rates in such patients are unknown. To the best of our knowledge, patients with heart valve replacement who were treated with RP have never been reported in the modern English medical literature. However, occasionally RP represents the treatment of choice for patients with a history of heart valve replacement. To address this knowledge gap, we relied on a population-based database of RP patients of whom some underwent heart valve replacement. We compared complication rates, length of stay, cost, and in-hospital mortality between RP patients with heart valve replacement and without heart valve replacement, and made several relevant observations.

First, a history of heart valve replacement in RP patients is rarely encountered. Specifically, only 0.3% of all RP patients previously underwent heart valve replacement. However, this proportion increased in a statistically significant fashion over the course of this study from 0.3% to 0.8% (*p* < 0.015). This observation indicates that the patients with heart valve replacement treated with RP are rare. However, the number of such patients is increasing. Therefore, it is important to provide reliable, evidence-based information on complication rates to patients with a history of heart valve replacement who are being considered for RP. These observations validate the need for the current study. Moreover, given the rarity of patients with heart valve replacement undergoing radical prostatectomy (0.3% in our cohort), these findings should be interpreted as preliminary. Future studies using external datasets, such as SEER-Medicare, are warranted to validate our results and potentially provide additional oncologic and long-term outcome data.

Second, we identified important differences in patient characteristics that distinguish RP patients with heart valve replacement from their RP counterparts without heart valve replacement. Specifically, RP patients with a history of heart valve replacement were older (median 66 vs. 62 years) than their respective counterparts and harbored an increasing proportion of patients with a history of heart valve replacement as CCI increased as follows: CCI 0–0.3%, CCI 1–0.4%, and CCI ≥ 2–0.7%. Conversely, the recorded rates of RP patients with heart valve replacement did not vary according to region, hospital size or teaching hospital status. These observations indicate that specific geographic locations are not exposed to higher rates of RP patients with heart valve replacement. Nonetheless, it is still essential to rely on multivariable adjustment for baseline characteristics since important differences persist regarding age and CCI status.

Third, we tested for differences in complication rates between RP patients with heart valve replacement versus their RP counterparts without heart valve replacement. Specifically, we focused on nine adverse in-hospital outcomes in separate, individually fitted multivariable models that addressed each of these outcomes of interest. In these analyses, a history of heart valve replacement was associated with higher rates of adverse in-hospital outcomes in eight of nine examined categories. Specifically, we recorded higher rates of postoperative bleeding (OR 16.2), cardiac complications (OR 3.9), infections (OR 3.7), use of CCT (OR 2.5), intraoperative complications (OR 1.9), blood transfusions (OR 1.5), longer hospital stay (RR 1.4), and higher estimated hospital cost (RR 1.1). Of these complications, postoperative bleeding, cardiac complications, and infections occupied the first, second, and third highest rank, respectively. They were followed by four adverse outcomes that exhibited an intermediate effect, namely: CCT use, intraoperative complications, and blood transfusions. The remaining variables, namely longer hospital stay, estimated hospital cost, and vascular complications, exhibited less pronounced effect. Nonetheless, they did achieve independent predictor status, except for vascular complications (*p* = 0.3). These findings are consistent with prior studies indicating that patients with cardiovascular comorbidities undergoing radical prostatectomy are at increased risk of postoperative complications. Siech et al. [3] reported that patients with ischemic heart disease had a significantly higher rate of postoperative infections and major cardiac events following RP. Our results extend these findings by focusing specifically on patients with prosthetic heart valve replacement, a subgroup previously unexplored in large population-level studies.

Fourth, we addressed in-hospital mortality which represents the ultimate complication. No statistically significant differences after RP were recorded in either univariable or multivariable analyses between patients with a history of heart valve replacement vs. others. Lack of in-hospital mortality rate differences between RP patients with a history of heart valve replacement vs. others is important in treatment decision-making and counseling when RP may represent a treatment option in patients with a history of heart valve replacement. Particularly, healthcare providers can reassure patients with a history of heart valve replacement that the risk of in-hospital mortality should not dissuade them from considering RP as a curative treatment option for prostate cancer when other options are less applicable. Moreover, although several other complications exhibited higher rates in RP patients with a history of heart valve replacement, these complications did not result in a substantial increase in the length of stay, nor did they result in a substantial increase in cost, despite the presence of statistically significant differences. Our findings align with evidence from Shan et al. [5], who showed that patients with cardiovascular comorbidities including heart disease may actually benefit from RP in terms of cardiovascular mortality compared to radiotherapy, especially in elderly men with localized disease. While our study focuses only on in-hospital outcomes, it reinforces the notion that RP remains a viable and safe curative treatment even in high-risk cardiovascular patients, provided appropriate perioperative management is ensured. To our knowledge, this is the first study specifically addressing RP safety in the subset of patients with prior heart valve replacement, thus filling a significant gap in the current literature.

Taken together, the prevalence of RP patients with a history of heart valve replacement is increasing over time in a statistically significant fashion. In consequence, it is imperative to objectively quantify complication profiles associated with RP in this select patient subgroup. The current analyses indicate that RP patients with heart valve replacement are at higher risk of complications than their RP counterparts without heart valve replacement. However, it appears that such complication profile is not prohibitive since it neither results in higher mortality nor in meaningful increases in length of stay or estimated hospital cost. This said, in rare situations when patients with a history of heart valve replacement may require RP, preoperative counseling and detailed informed consent should rely on the current results. This is essential to disclose complication rates most objectively, based on the best available evidence, with the intent of preoperative optimization of their medical condition and performance status.

The current study is not devoid of limitations. First, due to the retrospective nature of the NIS, selection and reporting biases may have remained. However, this limitation is shared with all previous analyses relying on the NIS [11,15,16,21] or other large-scale retrospective databases such as the Surveillance Epidemiology and End Results database [22]. Second, despite its very large size, NIS only provides for a limited number of patients with a history of heart valve replacement due to the rarity of this condition. Therefore, it was not feasible to carry out subgroup analyses categorized by the type of heart valve replacement: prosthetic vs. xenogenic vs. other. Since the vast majority of patients with a history of heart valve replacement treated with RP harbored prosthetic valve replacements, our findings are more generalized to this patient subgroup. Third, the NIS only offers a limited amount of detail. For example, timing, duration, and dose of anticoagulation were not available. Moreover, radical prostatectomy was selected as it remains a widely used curative option in localized prostate cancer. However, the NIS was unable to adjust for oncologic details (tumor stage, PSA levels, or histological grading) since the NIS does not contain such information. Fourth, the NIS exclusively provides in-hospital data and does not provide individual-level detail on specific comorbidities, limiting insight into which underlying conditions may contribute to adverse outcomes. In consequence, data regarding readmissions and complications after hospital discharge were not available. Indeed, it could be interesting to also assess readmission and long-term complications rates after RP in future studies. Additionally, although it is technically feasible to identify the surgical approach (open, laparoscopic, or robotic-assisted radical prostatectomy) using ICD procedure codes, this information is only consistently available from 2008 onward. Restricting our analysis to that time frame would have resulted in the exclusion of a large portion of the cohort, particularly affecting the already limited subgroup of patients with heart valve replacement. Therefore, to preserve statistical power and cohort integrity, the surgical approach was not included in the present analysis. Finally, NIS cost data are not linked to individual complications, precluding a detailed cost breakdown by complication type.

## 5. Conclusions

Following RP, patients with a history of heart valve replacement were at a higher risk for eight out of nine in-hospital outcomes, but not for higher in-hospital mortality. However, the small sample size in this group highlights the need for cautious interpretation of these findings.

## Figures and Tables

**Table 1:** Descriptive characteristics of prostate cancer patients undergoing radical prostatectomy stratified according to presence or absence of heart valve replacement.

Characteristic		Heart Valve Replacement, n = 694 (0.3%)	No Heart Valve Replacement, n = 219,664 (99.7%)	*p*-Value ^1^
**Age at admission**, median (IQR in years)		66 (61, 70)	62 (57, 67)	<0.001
**Charlson Comorbidity Index**, n (%)				<0.001
	0	425 (0.3%)	164,404 (99.7%)	
	1	165 (0.4%)	40,683 (99.6%)	
	≥2	104 (0.7%)	14,577 (99.3%)	
**Hospital region**, n (%)	West	152 (0.3%)	47,788 (99.7%)	0.01
	Midwest	189 (0.4%)	52,714 (99.6%)	
	Northeast	143 (0.4%)	40,274 (99.6%)	
	South	210 (0.3%)	78,888 (99.7%)	
**Teaching hospital**, n (%)		436 (0.3%)	138,275 (99.7%)	0.9
**Hospital size**, n (%)	Large (≥400 beds)	477 (0.3%)	149,200 (99.7%)	0.5
	Medium (200–399 beds)	138 (0.3%)	47,368 (99.7%)	
	Small (<200 beds)	79 (0.3%)	23,096 (99.7%)	

^1^ Wilcoxon rank sum test; Pearson’s Chi-square test; Fisher’s exact test. Abbreviations: IQR = Interquartile range.

**Table 2:** Perioperative complications, estimated hospital cost, length of stay, and mortality rates after radical prostatectomy in prostate cancer patients stratified according to presence or absence of heart valve replacement.

Characteristic	Heart Valve Replacement, n = 694 (0.3%)	No Heart Valve Replacement, n = 219,664 (99.7%)	*p*-Value ^1^
**Transfusion**, n (%)	73 (11%)	15,731 (7.2%)	<0.001
**Intraoperative complications**, n (%)	61 (8.8%)	9036 (4.1%)	<0.001
Intraoperative bleeding, n (%)	37 (5.3%)	2510 (1.1%)	<0.001
**Postoperative complications**			
Cardiac complications, n (%)	52 (7.5%)	2739 (1.2%)	<0.001
Bleeding, n (%)	<11 (<1.5%)	65 (<0.1%)	<0.001
Infections, n (%)	<11 (<1.5%)	221 (0.1%)	0.006
Vascular complications, n (%)	<11 (<1.5%)	561 (0.3%)	0.1
**Critical Care Therapy**, n (%)	<11 (<1.5%)	873 (0.4%)	<0.001
**Length of stay**, mean (SD, in days)	3.39 (3.42)	2.37 (2.03)	<0.001
**Estimated hospital cost**, median (IQR in $USD)	33,539 (22,715, 51,178)	30,716 (20,796, 45,307)	<0.001
**In-hospital mortality**, n (%)	<11 (<1.5%)	126 (<0.1%)	0.06

^1^ Wilcoxon rank sum test; Pearson’s Chi-square test; Fisher’s exact test. Abbreviations: SD = standard deviation, IQR = Interquartile range.

**Table 3:** Univariable and multivariable regression models predicting perioperative complications and in-hospital mortality according to presence or absence of heart valve replacement in prostate cancer patients undergoing radical prostatectomy after adjustment for clustering at the hospital level using generalized estimation equation (GEE) methodology.

	Univariable		Multivariable *	
Outcome of Interest	RR/OR (95% CI)	*p*-Value	RR/OR (95% CI)	*p*-Value
**Postoperative complications**				
Bleeding	19.6 (7.1, 54.1)	<0.001	16.2 (5.7, 46.3)	<0.001
Cardiac complications	6.2 (4.6, 8.2)	<0.001	3.9 (2.8, 5.3)	<0.001
Infections	5.5 (2.1, 14.4)	<0.001	3.7 (1.4, 9.8)	0.01
Vascular complications	2.2 (0.8, 5.9)	0.01	1.7 (0.6, 4.4)	0.3
**Critical Care Therapy**	3.5 (1.9, 6.5)	<0.001	2.5 (1.3, 4.6)	0.003
**Intraoperative complications**	2.1 (1.7, 2.8)	<0.001	1.9 (1.5, 2.5)	<0.001
Intraoperative bleeding	4.8 (3.4, 6.7)	<0.001	4.5 (3.2, 6.2)	<0.001
**Transfusions**	1.5 (1.3, 1.9)	<0.001	1.5 (1.2, 1.8)	<0.001
**Length of stay**	1.4 (1.3, 1.5)	<0.001	1.4 (1.3, 1.5)	<0.001
**Estimated hospital costs**	1.1 (1.1, 1.2)	<0.001	1.1 (1.0, 1.1)	<0.001
**In-hospital mortality**	4.9(1.2, 19.9)	0.023	2.8 (0.7, 11.4)	0.1

Reference: no history of heart valve replacement. * Adjusted for age at admission and comorbidities (Charlson Comorbidity Index). Abbreviations: CI = confidence interval; IRR = incidence rate ratio; OR = odds ratio.

## Data Availability

These data were derived from the National Inpatient Sample (NIS) database in the public domain: https://hcup-us.ahrq.gov/databases.jsp (accessed on 1 February 2024).

## Abbreviations

The following abbreviations are used in this manuscript:

RP	Radical Prostatectomy
NIS	National Inpatient Sample
HCUP	Healthcare Cost and Utilization Project
AHRQ	Agency for Healthcare Research and Quality
ICD-9-CM	International Classification of Disease, 9th Revision, Clinical Modification
ICD-10-CM	International Classification of Disease, 10th Revision, Clinical Modification
ICD-9-PCS	International Classification of Disease, 9th Revision, Procedure Coding System
ICD-10-PCS	International Classification of Disease, 10th Revision, Procedure Coding System
CCT	Critical Care Therapies
CCI	Charlson Comorbidity Index
EAPC	Estimated Annual Percentage Change
IQR	Interquartile Range
SD	Standard Deviation
OR	Odds Ratio
RR	Rate Ratio
CI	Confidence Interval
USD	United States Dollar
SEER	Surveillance, Epidemiology, and End Results (database)
